# Lemierre’s Syndrome Presenting with Multisystem Complications in a Child: A Case Report and Literature Review

**DOI:** 10.3390/reports8010007

**Published:** 2025-01-11

**Authors:** Ashwaq AlEed

**Affiliations:** Department of Pediatrics, College of Medicine, Qassim University, Buraydah 52571, Saudi Arabia; a.aleed@qu.edu.sa; Tel.: +966-163080500

**Keywords:** Lemierre’s syndrome, fever, stroke, infection, antibiotics, heparin

## Abstract

**Background and Clinical Significance:** Lemierre’s syndrome, also known as the “forgotten disease”, is a rare clinical syndrome of septic thrombophlebitis associated with morbidity and mortality. This study reports on a 6-year-old boy diagnosed with Lemierre’s syndrome, providing an in-depth case analysis and a comprehensive review of the current literature on this uncommon condition. **Case Presentation**: A 6-year-old boy was admitted to the pediatric intensive care unit (PICU) with septic shock, presenting with a high-grade fever of 39.5 °C for 10 days and swelling in the left leg for one week. Additionally, he had a history of swelling in the left mandibular area for five days and a skin rash. His recent medical history was unremarkable, except for decreased activity and oral intake over the past three days. Both his neonatal and past medical histories were unremarkable. Upon admission to the PICU, a multidisciplinary team was assembled to address his condition. Following a comprehensive history, physical examination, and relevant investigations, the child was diagnosed and managed as a case of Lemierre’s syndrome—the first reported case in Saudi Arabia. Treatment included antibiotics, unfractionated heparin infusion, and analgesics. Family members were counseled on the nature, severity, and prognosis of the disease. Despite the optimal treatment given to this patient, the patient died from multiorgan failure as a complication of the disease after an eight-day stay in the PICU. **Conclusions**: This paper reports the main presenting features and the workup of a 6-year-old male child diagnosed and managed as a case of Lemierre’s syndrome in the Saudi Arabian context. The early recognition of the symptoms of Lemierre’s syndrome and introduction of appropriate treatment in multidisciplinary teamwork are crucial to improve the outcomes of such a life-threating syndrome.

## 1. Introduction and Clinical Significance

Lemierre’s syndrome, also known as the “forgotten disease”, is a rare clinical syndrome of septic thrombophlebitis associated with morbidity and mortality [1]. The estimated worldwide incidence of Lemierre’s syndrome is 1/1,000,000 [2]. The literature shows that Lemierre’s syndrome is primarily the disease of healthy young adults, with a mean age at presentation of around 20 years old [1]. It can present in school-aged children, similarly to the present case [1,3]. Although *Fusobacterium necrophorum* is the most common isolated organism responsible for Lemierre’s syndrome [1,4,5], an increased trend of other organisms has recently been observed [4,6]. No previous data on Lemierre’s syndrome were published in Saudi Arabia. This study reports on a 6-year-old male child from Saudi Arabia diagnosed with Lemierre’s syndrome and reviews the existing literature on the condition.

## 2. Case Presentation

A 6-year-old male child with up-to-date vaccinations was apparently healthy before this episode. He was brought to the hospital by his mother due to complaints of a high-grade fever of 39.5 °C for 10 days and swelling of the left leg for one week. He had a history of swelling in the left mandibular area for five days and a skin rash, as well as a history of decreased oral intake and activity for three days. He was drowsy and had not been ambulatory for the last 24 h. He had no history of vomiting, diarrhea, cough, or shortness of breath, and no history of contact with pets/animals or sick patients or change in urine color or smell. There was no history of trauma and no headache, blurring of vision, blood in stool, abdominal pain, a yellowish discoloration of sclera, weight loss, or loss of appetite. Past Medical and Surgical History: The patient had no history of surgical operation, abscess drainage, or blood transfusion. He had no known allergies to medication and was not on any regular medication/steroids/immune suppressants/immune modulators. Neonatal History: Normal pregnancy with normal vaginal delivery with no neonatal intensive care unit (NICU) admission. The patient had a normal developmental history. He consumed a normal diet with no raw milk ingestion. On Emergency Department Examinations, regarding Vital Signs, the patient’s heart rate was 186/min, oxygen saturation was 94%, and blood pressure (BP) was 110/69 mmhg. He was tachycardic. He had a high fever of 39.5 °C and capillary refill time of 3 s. Central Nervous System: The patient was drowsy. His Glasgow Coma Scale (GCS) score was 8/15. The central nervous system examination revealed no signs of meningeal irritation. His pupils were normal in size and showed normal reactions (B/lNsrl). Ears, Nose, and Throat: Normal. Respiratory examination: The patient had noisy breathing but good air entry bilaterally with bilaterally conducted sounds. Cardiology: The patient had heart sounds of S1 + S2 + 0. Abdomen: The patient’s abdomen was soft, with mild fullness, and hepatomegaly was 3 cm below the costal margin. Skin: The patient had a petechial rash over his face. An abrasion was noted over his upper lip. He also showed swelling in the left mandibular area that extended posteriorly to the mastoid region, with redness over the skin and tenderness, suggesting submandibular lymphadenitis.

### Investigations

Complete Blood Count: The patient’s white blood cell count (WBC) was 19.9 × 10^9^/L, the C-reactive protein value was 338 mg/L, the ESR value was 34 mm/hr, and the procalcitonin value was 13.6 ng/mL. The coagulation profile was checked prior to the procedures and was normal. A venous blood gas (VBG) analysis showed pH, 7.38; PCo2, 39 kPa; HCO3, 23 mmol/L; K, 3.3 mEq/L; Na, 161 mmol/L; Cl, 132 mEq/L; and hemoglobin, 7.9 mg/dL.

**Figure 1:**
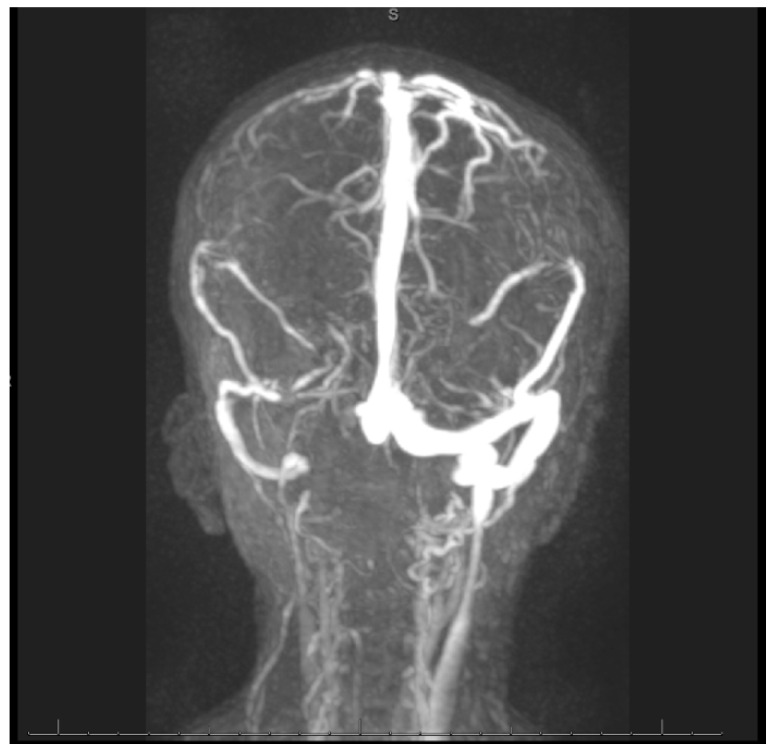
Brain and neck computed tomography with contrast.

**Figure 2:**
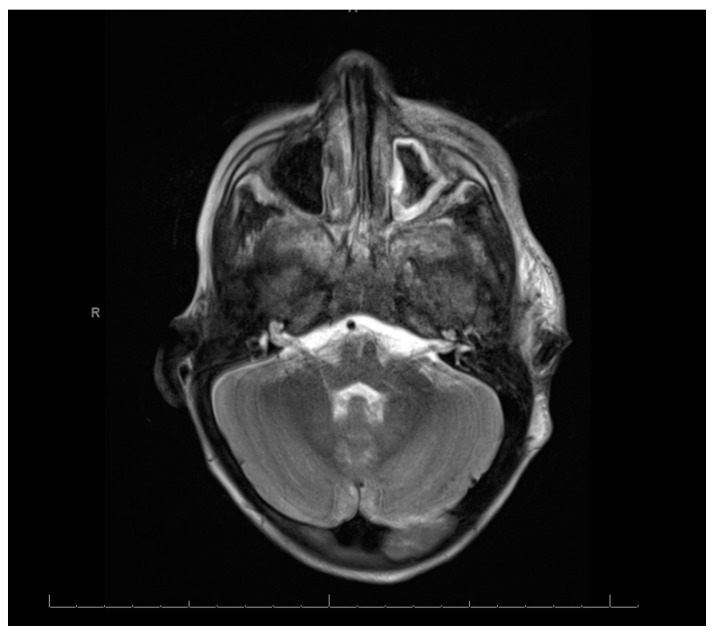
Brain and neck computed tomography with contrast (cross-sectional view).

Swabs: A nasopharyngeal swab for a viral multiplex polymerase chain reaction (PCR) was negative. A nasopharyngeal swab for coronavirus disease was negative. A wound swab of the pus aspirate from the oral cavity revealed Gram-negative rods found to be Fusobacterium necrophorum. The identification and susceptibility of causative microorganisms were performed by a VITEK 2 compact system and antibiotic susceptibility testing was interpreted using internationally recognized standards, those established by the Clinical and Laboratory Standards Institute (CLSI) [7]. Cerebrospinal Fluid (CSF): The CSF was clear and colorless. The total WBC was 72 /mm^3^. The neutrophil content was 94%, the lymphocyte content was 4%, and the monocyte content was 2%. The level of protein in the CSF was high at 0.8 mg/dL, and the glucose value was 2.2 mg/dL. The patient’s gum pus was positive for methicillin-resistant Staphylococcus aureus (MRSA). A blood culture showed Gram-positive cocci in clusters. However, there was no history of any family member with MRSA and the patient was not admitted to the hospital before this admission. Echocardiography: Normal. Electroencephalogram: The electroencephalogram indicated a subclinical seizure. Brain and neck computed tomography (CT) with contrast findings is shown in Figure 1 and Figure 2.

Figure 1 and Figure 2: (A) Loss of corticomedullary differentiation in the right frontal, parietofrontal, and left parietal lobes, indicating a possible microinfarction. (B) Thrombosis in the right internal jugular vein. (C) Left maxillary and ethmoidal sinusitis. The neck CT showed the following: (A) An enlarged heterogenous left parotid gland containing multiple micro-abscesses, with soft tissue swelling and cervical lymphadenopathy, which could represent an infectious/inflammatory process. (B) Right internal jugular vein thrombosis. (C) Multiple bilateral peripheral lung nodules, suggesting that the differential diagnosis could include septic emboli.

Magnetic Resonance Imaging (MRI), Magnetic Resonance Angiography (MRA), and Magnetic Resonance Venography (MRV): Magnetic resonance protocols indicated high T2 signal intensities extensively in the bilateral frontal, parietal, and occipital cortical and subcortical areas, with diffusion restriction. These findings were compatible with acute watershed infarctions with multifocal areas of embolic infarcts, suggestive of severe hypoxic–ischemic encephalopathy. The differential diagnosis included sepsis/septic shock and vasculitis, supporting a stroke workup. The right internal carotid long segment showed severe narrowing/occlusion, and the left internal carotid short segment showed narrowing. The differential diagnosis included vasculitis and sepsis/septic shock with dissection.

The right transverse sinus and internal jugular vein showed thrombosis.

MRA images: The images demonstrate a severe narrowing/occlusion of a long segment of the right internal carotid artery and a short segment of narrowing along the left internal carotid artery. Major intracranial cerebral arteries were patent. MRV images: The images demonstrate an occlusion of the proximal right transverse sinus and narrowing of the right internal jugular vein. The remaining superficial and deep cerebral veins and dural venous sinuses were patent.

**Figure 3:**
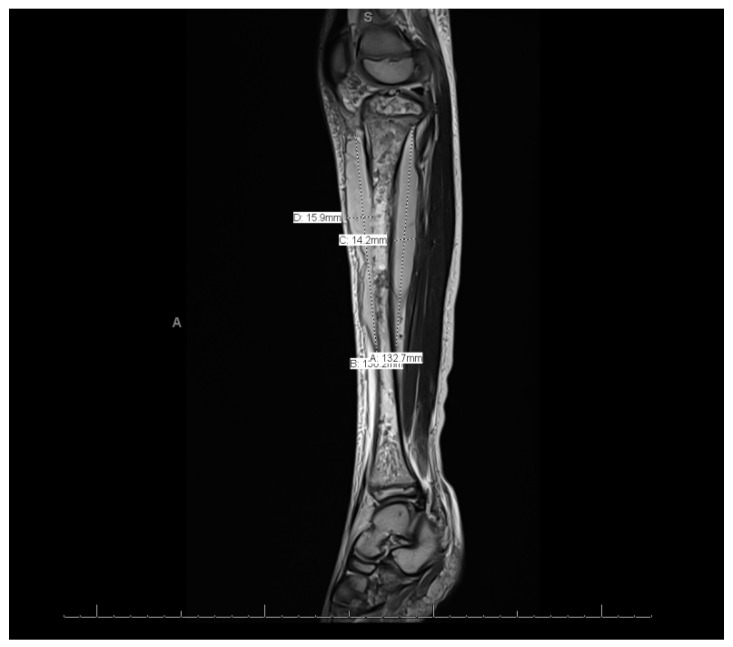
Magnetic resonance imaging of the whole left leg.

**Figure 4:**
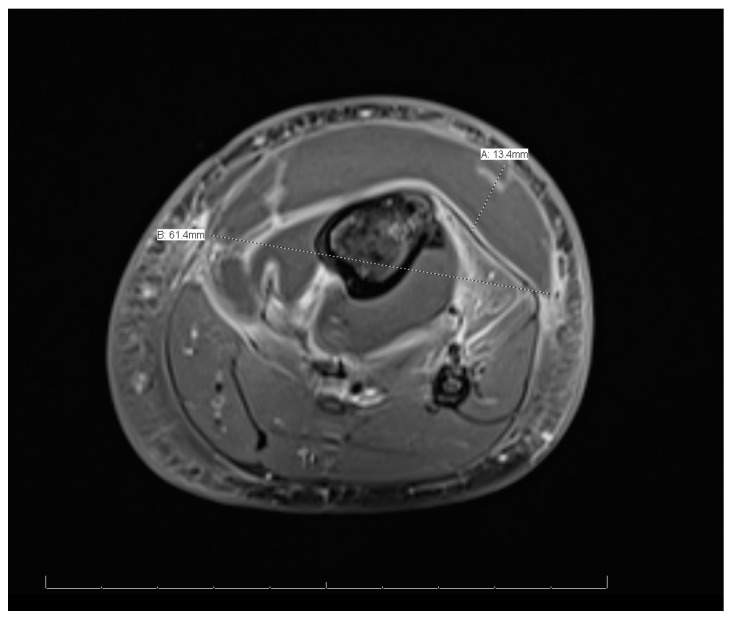
Magnetic resonance imaging of the whole left leg (cross-sectional view).

MRI of the left leg (Figure 3 and Figure 4): The whole left tibial bone shaft (proximal, mid, and distal), including the proximal epiphysis, showed a diffuse, multiple, heterogeneous marrow signal, intermediate to low on T1 and high on T2, as well as bone marrow edema associated with a periosteal reaction seen mainly on the medial aspect of the proximal metaphysis of the tibia. This is a large lobulated/septated periosteal collection surrounding all aspects of the proximal region and the mid-shaft of the left tibia bone. The collection ran along posteriorly for a distance of 13.3 cm × 3.2 cm × 1.4 cm (cranio-caudal [CC], medial–lateral [ML], and anterior–posterior [AP], respectively), with a small pocket extending to the posterior muscle compartment involving the knee joint, which showed joint effusion and synovitis. The anterior part of the collection extended partially to the anterior subcutaneous area and measured 13 × 5.7 × 1.6 (CC, ML, and AP, respectively). Both the anterior and posterior parts of the collections were connected. A focal cortical defect was evident at the anterior medial aspect of the proximal tibial epiphysis (T2 STIR axial 15/44 image), with an adjacent periosteal collection. The fibula, femur, and ankle joint were not involved and showed a normal signal. Diffuse soft tissue swelling was evident, mainly in the proximal part of the leg. The muscles around the leg and knee joint appeared to be normal. The MRI findings were in line with acute osteomyelitis of the left tibia, with periosteal collections.

**Treatment Plan:** The patient was started on a normal saline (NS) bolus at 20 mL/kg over 20 min. The NS bolus was repeated in the same volume due to prolonged capillary refill time. The patient received ceftriaxone (1 g i.v. stat), i.v. clindamycin (10 mg/kg), and i.v. vancomycin (20 mg/kg). An unfractionated heparin infusion at 400 U/h was initiated by the hematology team. The family members were counseled about the nature of the disease, its severity, and its prognosis. In the pediatric intensive care unit (PICU), the treatments were adjusted based on the patient status and investigation results. The patient was intubated and connected to a mechanical ventilator due to decreased O2 saturation. The patient developed several complications, including a sudden attack of hypotension and hypothermia, decreased O2 saturation, acute renal failure, and pneumothorax with pleural effusion. Despite optimal treatment given to this patient, the patient died from complications of the disease after an eight-day stay in the PICU.

## 3. Discussion

This study addressed a case scenario of a 6-year-old child presented to a pediatric emergency department. After a thorough history taking, physical examination, and appropriate investigations, including blood culture, CT, and MRI, the child was diagnosed and managed as a case of Lemierre’s syndrome. The literature shows that Lemierre’s syndrome is primarily the disease of healthy young adults, with a mean age at presentation of around 20 years [1]. It can be present in school-aged children, similarly to this case [1,3]. For example, Ibrahim et al. in the United Arab Emirates (UAE) reported a case of a 6-year-old boy who presented with fever and headache with a history of sore throat [3]. The high-grade fever in this case (39.5 °C) is consistent with the presentation in other previous cases, including that in Saudi’s neighboring country, the UAE (39.0 °C) [3,8]. The large heterogeneity of Lemierre’s syndrome from case to case may pose great difficulty in achieving timely clinical diagnoses. Although this reported case was similar in clinical presentation to other reported cases, Lemierre’s syndrome can present in different clinical presentations, such as cranial nerve paralysis [5].

Similarly to other studies, our case showed thrombosis in the right transverse sinus and internal jugular vein [4,6]. In Salami et al.’s case series (eight patients), internal jugular vein thrombosis was more common than bilateral thrombosis, especially the right internal jugular vein (seven cases vs. one case) [6]. The presence of thrombosis in the right internal jugular vein rather than the left one could be explained by the differences in anatomy and blood flow stasis.

Although various organisms were isolated by culture, the most commonly isolated one was Fusobacterium necrophorum [1,4,5]. This case showed a positive culture of Gram-negative bacilli bacteria in the oral cavity, MRSA in the gums, and Gram-positive cocci in clusters in blood culture. Therefore, these isolated organisms support the current trend of increasing the isolation of other organisms [1]. In support of this, Sallami et al. reported that *F. necrophorum* was not found in any of the case series (n = 8) [6]. Recently, in China, *F. necrophorum* was detected by plasma using metagenomic next-generation sequencing (mNGS) testing [5]. This indicates that mNGS may play an important role in the rapid and accurate pathogenic diagnosis of Lemierre’s syndrome and thus in implementing accurate and effective management [5]. Negative cultures of organisms have also been reported in the literature [4], and this cannot rule out the presence of Lemierre’s syndrome. Negative cultures may indicate the early introduction of antibiotics before sample taking [6], or more sensitive tools are needed to detect even small numbers of organisms [5].

The choices of antibiotics for this patient (i.e., ceftriaxone, clindamycin, and vancomycin) are in accordance with a recent published meta-analysis [4]. The high mortality rate of this syndrome (as high as 90%) before the era of antibiotics should be emphasized [1,2,3]. Researchers predict the re-emergence of Lemierre’s syndrome in the near future due to bacterial antibiotic resistance and a greater reluctance to prescribe antibiotics for minor conditions, such as tonsillitis [1,9]. This antibiotic resistance could contribute to the increasing incidence of Lemierre’s syndrome mortality in the future. Therefore, the rational and appropriate use of antibiotics should be strategic.

This case showed distant metastatic emboli to the left leg. Other studies showed different sites, such as the lungs [10]. Similarly to other cases, this case was treated without surgical interventions because there was no obvious surgical necrosis that necessitated debridement, and cases requiring surgical interventions are rare [10]. The sign that indicated migration was that swelling of the left leg began after the fever onsite. In addition, the imaging modalities for this patient, including MRI, indicated migration.

Similarly to other reported cases, our patient received anticoagulants (i.e., unfractionated heparin) [4,9]. The role of anticoagulants in the management of Lemierre’s syndrome is unclear [11]. This controversy in using anticoagulants in the management of Lemierre’s syndrome requires further sophisticated research to explore cases of Lemierre’s syndrome that respond to anticoagulants. A composition of the present literature on cases of anticoagulants per vessel involved, type of organism, and type of anticoagulant itself could be a good approach for future research. In addition, conducting international randomized trials could solve this controversy and investigate the mechanism of anticoagulant therapy in Lemierre’s syndrome. Coultas et al. reported using and not using anticoagulation therapy to determine the benefits and risks, with close monitoring of the patient’s bleeding profile and the anticipation of the interaction of certain anticoagulation therapies, such as warfarin with metronidazole [9]. For example, Ibrahim et al. suggested using anticoagulation therapy when the thrombosis propagates retrogradely and involves the cavernous sinuses of the brain [3]. Conversely, in Santos et al.’s study, anticoagulant therapy was not performed on their 31-year-old male patient, who achieved full clinical recovery, due to the risks and the lack of clear benefits described in the existing literature [12]. A recent meta-analysis reported no statistically significant effect on vessel recanalization or mortality in patients treated with anticoagulation versus those who were not [4]. In their case series, Salami et al. found that the choice of surgical debridement and anticoagulation therapy depends on its justification in certain specific situations; anticoagulant therapy was justified in three out of eight patients, and no deaths were reported in both groups [6].

Moreover, antibiotics, anticoagulant therapy, hydration with NS, and analgesics were given to this patient. Such supportive care measures are recommended when there is a need for them [13]. To ensure that the patient received appropriate antibacterial treatment, antibiotics were administered according to the sensitivity results.

As previously mentioned, this patient developed several complications, which required further interventions, including intubation and mechanical ventilation. Such complications have been reported to be associated with Lemierre’s syndrome, especially those related to lungs [2]. The negative outcome of this case indicates the high mortality rate of this life-threatening disease. Despite the presence of antibiotics, the mortality rate of Lemierre’s syndrome remains high, especially among those who seek healthcare late and with complications [4,5]. An indication that the present patient sought healthcare services late was a low GCS score of 8/15 at presentation. In Saudi Arabia, a low GCS score is the most important prognostic factor for outcomes, especially in children with non-traumatic comas, including septic shock [14].

Allen et al. reported that, even with appropriate antibiotics and therapy, the mortality rate of Lemierre’s syndrome ranged from 5% to 18% [2]. This high mortality rate is common among patients with septic emboli and end-organ damage [2]. Moreover, a recent meta-analysis conducted by Gore revealed a 4.1% mortality rate of Lemierre’s syndrome—that is, 16 deaths out of 394 patients were reported [4].

To the best of the author’s knowledge, this case of Lemierre’s syndrome, also known as the “forgotten disease”, is the first reported in Saudi Arabia. This case report adds to the literature addressing Lemierre’s syndrome, the early recognition of its symptoms and signs, and its appropriate management to avoid catastrophic complications for better child health [1]. Although the outcome was negative despite optimal treatment, this study highlights the importance of adopting the multidisciplinary team approach to avoid a misdiagnosis and delays in the diagnosis and management of such a potentially fatal disease.

## 4. Conclusions

In conclusion, this case report discusses a pediatric patient diagnosed with Lemierre’s syndrome, a rare but life-threatening condition often resulting from a throat infection that leads to septic thrombophlebitis of the internal jugular vein. The child presented with classic symptoms, including severe fever and neck swelling. The rapid progression of the illness necessitated immediate medical intervention. However, despite aggressive treatment, the child succumbed to complications of Lemierre’s syndrome. The novelty of this case lies in the atypical presentation and rapid deterioration of the patient, highlighting the challenges in early diagnoses and management of Lemierre’s syndrome in pediatric patients. This case underscores the importance of considering Lemierre’s syndrome in pediatric patients presenting with pharyngitis and systemic symptoms, even when typical signs may not be immediately apparent. Clinically, this case emphasizes several key implications for healthcare professionals. First, it highlights the urgent need to raise awareness of Lemierre’s syndrome, particularly in pediatric settings. Early recognition and timely intervention are essential for improving outcomes. Additionally, this report advocates for the implementation of protocols to rapidly diagnose and manage suspected cases of Lemierre’s syndrome, including the use of imaging studies and appropriate antibiotic therapy guided by sensitivity patterns. This case serves as a vital reminder of the potential severity of seemingly mild fever and benign throat infections in children, underscoring the need for vigilance in monitoring their progression. The main lesson learned by our pediatric department is the importance of establishing a holistic, multidisciplinary approach to manage such cases in the future. Despite the unfortunate outcome in this case, which is consistent with previous reports, it emphasizes the need for heightened awareness, early diagnoses, and effective management strategies to improve patient care [4]. The early recognition of the symptoms of Lemierre’s syndrome and working as a multidisciplinary team are crucial in improving such life-threatening cases.

## Data Availability

The data supporting the current study’s findings will be available upon rational request from the author. The data are not publicly available due to privacy concerns.
